# Deviated Uvula Resulting From Herpes Zoster Infection: A Case Report

**DOI:** 10.7759/cureus.103600

**Published:** 2026-02-14

**Authors:** Ramachandra Reddy Gowda Venkatesha, Karthik Rajaram Mohan, Sindhuja Rajalingam, Saramma Mathew Fenn, R. T. Reethika Rathan

**Affiliations:** 1 Department of Oral Medicine and Radiology, Vinayaka Mission's Sankarachariyar Dental College, Vinayaka Mission's Research Foundation (Deemed to be University), Salem, IND

**Keywords:** cranial nerve paralysis, uvula, varicella zoster (chicken pox), varicella zoster virus infection, herpes zoster

## Abstract

The uvula is a soft tissue that freely hangs down from the soft palate posterior margin. It prevents the soft palate from entering the nasopharynx during sneezing and coughing. The uvula contains mucus glands, which help to keep the throat moist and facilitate swallowing. The uvula plays a vital role in speech and helps deflect foreign bodies accidentally entering the oral cavity by triggering the gag reflex on touch. Deviation of the uvula is often an overlooked sign by oral physicians. Deviation of the uvula is linked to viral illnesses such as herpes zoster (HZ), coronavirus disease 2019, obstructive sleep apnea, and peritonsillar abscesses. It also occurs in vagus nerve palsy secondary to HZ infection. The case report describes such an occurrence of hockey-stick-shaped swollen, deviated uvula in a 28-year-old male patient, clinical features, differential diagnosis, and preventive vaccines described here.

## Introduction

Herpes zoster (HZ) from the varicella-zoster virus (VZV) also affects the vagus nerve but is uncommon [1,2]. VZV is a neurotropic alpha-herpesvirus in humans that, after initial infection, becomes latent in neurons within the peripheral autonomic enteric ganglia [2]. In particular, neurons in the cranial nerve ganglia and dorsal root ganglia are infected by the HZ virus [2]. VZV stays dormant in these ganglia's neurons following the initial infection (chickenpox). Reactivation results in HZ, often known as shingles, which causes discomfort and dermatomal rashes due to inflammation and nerve damage. Although VZV is mainly neuroinvasive in the peripheral nervous system, it can also have neurovirulent effects, resulting in serious nervous system diseases like postherpetic neuralgia and, in rare cases, complications in the central nervous system like myelitis [2]. Varicella (chickenpox) infection results in HZ virus infection [2]. The HZ virus remains latent in the dorsal root ganglia. Reactivation is more familiar with reduced or impaired cell-mediated immunity [1-3]. Deviation of the uvula is also reported as a complication of local anesthetic infiltration during dental rehabilitation in pediatric patients [3]. HZ may be overlooked early in patients of abducens nerve palsy with atypical vesicular lesions, and thus, a systemic evaluation is of extraordinary importance in the diagnosis [3]. The prognosis of vagus nerve palsy is good but poor in the case of vagus nerve palsy associated with another cranial nerve. This glossopharyngeal nerve increases the risk of complications like dysphagia or aspiration pneumonia [4]. Studies have discovered certain elements in the VZV genome, like open reading frame 7 (ORF7), that support the virus's neurotropic characteristics by promoting viral replication and dissemination in neural tissues [5]. The protein this gene encodes is essential to the neurotropism of the VZV, which causes shingles and chickenpox [5]. A crucial component of the virus's capacity to infect neurons and produce neurological symptoms, ORF7 is found in the Golgi compartment of infected cells [5]. It is necessary for viral dissemination in nervous tissue [5]. The deviated uvula can result in complications like obstructive sleep apnea during sleep, dysphagia, and speech intelligibility (ability to understand words) [6].

## Case presentation

**Figure 1:**
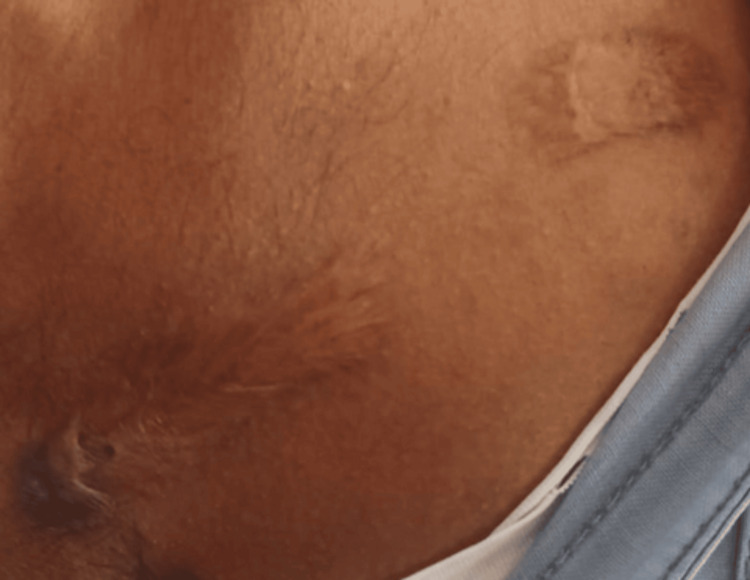
Examination revealing scars on the chest

A 28-year-old male patient reported for a routine dental checkup. History revealed the patient had an episode of chickenpox (varicella zoster) infection during his childhood days. He had no habits of chewing or smoking tobacco or alcohol consumption. General examination revealed his vitals were stable. No history of nasal blocks, congestion, or snoring while sleeping at night ruled out obstructive sleep apnea in our patient. An extraoral examination revealed scars on the chest (Figure 1).

**Figure 2:**
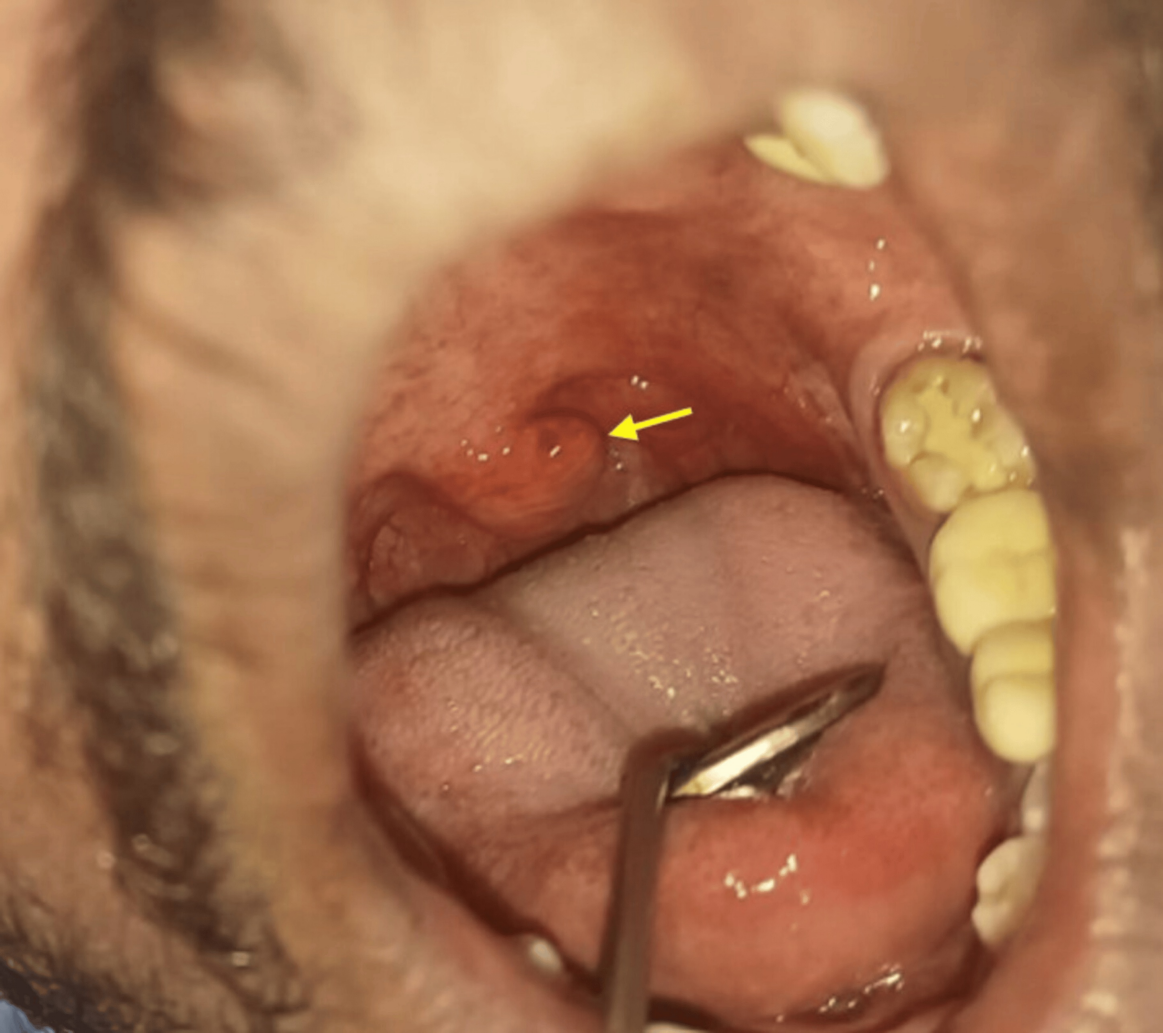
Intraoral examination revealing hockey-stick-shaped deviated uvula (yellow arrow)

Intraoral examination revealed a deviated left-side uvula like a hockey stick (Figure 2).

The differential diagnosis includes oral submucous fibrosis involving the uvula, peritonsillar abscess, and advanced stages of oral submucous fibrosis. In advanced stages of oral submucous fibrosis, the uvula appears constricted, small, shrunken like a bud, and deviated like a hockey stick due to progressive fibrosis. In our case, the size of the uvula appeared larger. Infection in the peritonsillar area (Quinsy) causes a deviated uvula. No redness or abnormal discharge was appreciated in the peritonsillar area. In the examination of the tongue on protrusion, there was no deviation of the tongue; this ruled out hypoglossal cranial nerve palsy (Video 1).

**Video 1:** Examination of tongue revealing no deviation of tongue on protrusion

**Figure 3:**
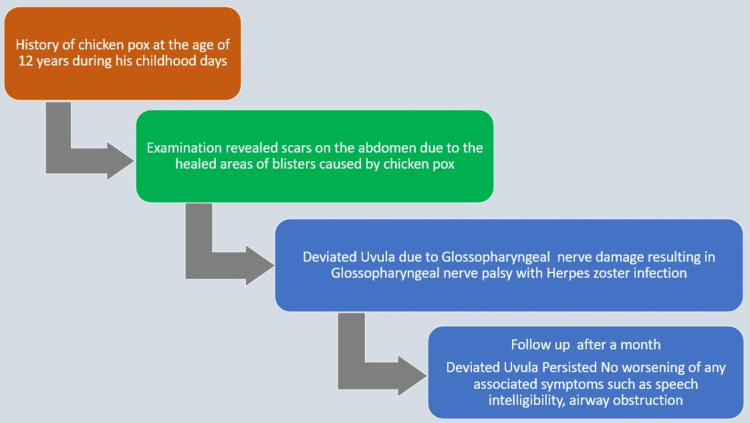
A brief timeline of history

 A brief timeline of history is mentioned in Figure 3.

The antiviral drug acyclovir 800 mg every four hours, five times a day for 7-10 days, is recommended within 48-72 hours of the onset of clinical symptoms of HZ. The patient's condition is asymptomatic with no associated symptoms such as speech intelligibility or airway obstruction due to a deviated uvula, which increases the risk of obstructive sleep apnea during sleep. A month's follow-up did not worsen or improve the clinical signs of deviated uvula. The weakness caused by glossopharyngeal nerve damage due to HZ infection led to the persistence of the deviated uvula.

## Discussion

HZ from the VZV also affects the vagus nerve but is uncommon [2]. VZV is a neurotropic alpha-herpesvirus in humans that, after initial infection, becomes latent in neurons within the peripheral autonomic enteric ganglia. Reactivation can be spontaneous or upon eliciting factors if cell-mediated immunity declines. Varicella (chickenpox) infection results in HZ virus infection. The HZ virus remains latent in the dorsal root ganglia. Reactivation is more familiar with reduced or impaired cell-mediated immunity. Deviation of the uvula is also reported as a complication of local anesthetic infiltration during dental rehabilitation in pediatric patients [3]. The prognosis of vagus nerve palsy is good but poor in the case of vagus nerve palsy associated with another cranial nerve. This glossopharyngeal nerve increases the risk of complications like dysphagia or aspiration pneumonia [4]. The ORF7 gene is implicated in the transmission and multiplication of the virus through capsids and causes nerve damage to infected neuron cells [5]. In children and adolescents, HZ is uncommon and related to cancer [6].

HZ may be overlooked early in patients of abducens nerve palsy with atypical vesicular lesions, and thus, a systemic evaluation is of extraordinary importance in the diagnosis [6]. A study found that the incidence rate of HZ was 20.7 cases with an incidence rate ratio of 8.6 (20.7 cases per 10,000 person-years with cancer to 2.4 per 10,000 healthy children), highlighting a significant association between childhood cancer and HZ risk [6]. The most common risk factors in adults include old age, stress, and infections such as coronavirus disease 2019 (COVID-19), acquired immunodeficiency syndrome, and conditions that cause immunosuppression diabetes. During the COVID-19 pandemic era, HZ reactivation has been reported after COVID-19 vaccination. The disease presents various clinical stages of variable clinical manifestations. Some of the manifestations carry a greater risk of complications [7].

Following a primary infection, VZV and herpes simplex virus (HSV) are alpha-herpes viruses that cause latent, lifelong infection within neuronal ganglia. These viruses are periodically reactivated to give rise to recurrent infections that significantly affect the quality of life. HSV infects genital and oral mucocutaneous tissues, while VZV causes varicella/chickenpox and HZ/shingles [8].

The prelicensure clinical trials report the efficacy of the live zoster vaccine as 50%-70% and that of the recombinant vaccine to be higher at 90%-97%. The effectiveness of vaccines was 85% and 46% for the recombinant and live zoster vaccines, respectively. Rare side effects that occur in less than 1% of cases, such as disseminated HZ with the live zoster vaccine and Guillain-Barré syndrome, are also associated with the recombinant zoster vaccine (RZV) [9]. The RZV and zoster live vaccine are approved to prevent postherpetic neuralgia and HZ [10]. HZ results from the reactivation of latent VZV infection in sensory (cranial, dorsal root) ganglia [11]. Immunosuppression and older age are the key risk factors for HZ [11]. HZ ophthalmicus is a form of HZ that involves the ophthalmic division of the trigeminal or fifth cranial nerve [11]. Ocular disease manifestations are keratitis, conjunctivitis, and uveitis, while retinitis and optic neuropathy are rare. Because of the risk of visual impairment, ocular involvement needs emergent evaluation by an ophthalmologist. Prompt diagnosis and early treatment within 72 hours with antivirals can avert ocular complications [11].

The incidence and recurrence rates of HZ were about 0%-18.2% and 17-55 per 1,000 person-year in immunocompromised individuals, respectively [12]. Comorbid states such as diabetes increase the risk of HZ [12]. The increase in HZ infections was higher during the COVID-19 pandemic [13]. Edematous, deviated uvula may also occur due to COVID-19 infection from uvulitis [13]. In oral submucous fibrosis of the uvula, the uvula is shortened by fibrosis, which is deviated like a hockey stick. In our patient, the uvula was deviated like a hockey stick and edematous [14]. In hypoglossal nerve palsy, the tongue deviates to the affected side on protrusion of tongue [15]. During tongue examination, his tongue did not deviate on protrusion; hypoglossal nerve palsy was ruled out. Since no other associated symptoms, such as sore throat, dysarthria, or sleep disturbance, were noted, no treatment was indicated in our case. The deviation of the uvula can also result from peritonsillar abscess [16]. Uvular hydrops is a self-limiting condition characterized by a swollen uvula, usually associated with allergy [16]. The VZV affects the vagus, abducent, facial, and glossopharyngeal nerves, resulting in polyneuropathies [17].

## Conclusions

A systemic clinical examination is always essential in patients with deviated uvula to rule out viral infections such as HZ-induced vagus nerve palsy resulting in deviated uvula. Vagus nerve palsy secondary to HZ is an uncommon but established complication where the vagus nerve is the target of the VZV infection. Diagnosis can be difficult and may need systemic assessment, high suspicion, and supporting evidence like polymerase chain reaction for VZV DNA. Initiation of antiviral treatment early is vital to prevent nerve damage and optimize outcomes, though the prognosis for complete recovery of vagal nerve function is guarded. In cases of cranial nerve palsy without the characteristic vesicular lesions, HZ should be included in the differential diagnosis based on the clinical presentation.
